# Phenome-Wide Association Study of Latent Autoimmune Diabetes from a Southern Mexican Population Implicates rs7305229 with Plasmatic Anti-Glutamic Acid Decarboxylase Autoantibody (GADA) Levels

**DOI:** 10.3390/ijms251810154

**Published:** 2024-09-21

**Authors:** Germán Alberto Nolasco-Rosales, José Jaime Martínez-Magaña, Isela Esther Juárez-Rojop, Ester Rodríguez-Sánchez, David Ruiz-Ramos, Jorge Ameth Villatoro-Velázquez, Marycarmen Bustos-Gamiño, Maria Elena Medina-Mora, Carlos Alfonso Tovilla-Zárate, Juan Daniel Cruz-Castillo, Humberto Nicolini, Alma Delia Genis-Mendoza

**Affiliations:** 1 División Académica de Ciencias de la Salud, Universidad Juárez Autónoma de Tabasco, Villahermosa 86100, Mexico; ganr_1277@live.com.mx (G.A.N.-R.); iselajuarezrojop@hotmail.com (I.E.J.-R.); daruiz_914@hotmail.com (D.R.-R.); juandaniel881@gmail.com (J.D.C.-C.); 2 Department of Psychiatry, Yale School of Medicine, Yale University, New Haven, CT 06520, USA; jose.martinez-magana@yale.edu; 3 VA Connecticut Healthcare System, West Haven, CT 06516, USA; 4 U.S. Department of Veterans Affairs National Center for Posttraumatic Stress Disorder, Clinical Neurosciences Division, West Haven, CT 06516, USA; 5 Hospital Regional de Alta Especialidad “Dr. Gustavo A. Rovirosa Pérez”, Secretaría de Salud, Villahermosa 86020, Mexico; esters56@hotmail.com; 6 Instituto Nacional de Psiquiatría Ramon de la Fuente Muñiz, Secretaría de Salud, Mexico City 14370, Mexico; ameth@inprf.gob.mx (J.A.V.-V.); naomi_gam@hotmail.com (M.B.-G.); metmmora@gmail.com (M.E.M.-M.); 7 Facultad de Psicología, Universidad Nacional Autónoma de México—UNAM, Mexico City 04510, Mexico; 8 División Académica Multidisciplinaria de Comalcalco, Universidad Juárez Autónoma de Tabasco, Villahermosa 86658, Mexico; alfonso_tovillaz@yahoo.com.mx; 9 Laboratorio de Enfermedades Psiquiátricas, Neurodegenerativas y Adicciones, Instituto Nacional de Medicina Genómica, Secretaría de Salud, Mexico City 14610, Mexico

**Keywords:** latent autoimmune diabetes of adults, genome-wide association study, Mexico, fas apoptotic inhibitory molecule 2

## Abstract

Latent autoimmune diabetes in adults (LADA) is characterized by the presence of glutamate decarboxylase autoantibodies (GADA). LADA has intermediate features between type 1 diabetes and type 2 diabetes. In addition, genetic risk factors for both types of diabetes are present in LADA. Nonetheless, evidence about the genetics of LADA in non-European populations is scarce. This study aims to perform a genome-wide association study with a phenome-wide association study of LADA in a southeastern Mexican population. We included 59 patients diagnosed with LADA from a previous study and 3121 individuals without diabetes from the MxGDAR/ENCODAT database. We utilized the GENESIS package in R to perform the genome-wide association study (GWAS) of LADA and PLINK for the phenome-wide association study (PheWAS) of LADA features. Nine polymorphisms reach the nominal association level (1 × 10^−5^) in the GWAS. The PheWAS showed that rs7305229 is genome-wide and associated with serum GADA levels in our sample (*p* = 1.84 × 10^−8^). rs7305229 is located downstream of the FAIM2 gene; previous reports associate FAIM2 variants with childhood obesity, body mass index, body adiposity measures, lymphocyte CD8+ activity, and anti-thyroid peroxidase antibodies. Our findings reveal that rs7305229 affects the GADA levels in patients with LADA from southeastern Mexico. More studies are needed to determine if this risk genotype exists in other populations with LADA.

## 1. Introduction

The World Health Organization estimates that 5–14% of patients with diabetes have latent autoimmune diabetes in adults (LADA) [1,2]. It is described that subjects with LADA have intermediate features between type 1 diabetes (T1D) and type 2 diabetes (T2D). LADA is characterized by slower autoimmune destruction of the β-cells compared to T1D, where glutamate decarboxylase autoantibodies (GADA) are the predominant serum marker [3,4]. This autoimmune process results in progressive hyperglycemia, and thus, patients with LADA do not require insulin therapy at the onset of diabetes [5]. On the other hand, the etiology of LADA involves insulin resistance. As with T2D, the risk of LADA is modified by lifestyle factors such as body mass index and physical activity [6]. Moreover, the initial clinical presentation of LADA resembles that of T2D. Consequently, patients with LADA are initially diagnosed with T2D, and they receive inadequate therapies for their diabetes until insulin dependence develops [5,7].

Buzzetti et al. (2020) suggest that the phenotype of LADA is heterogeneous with some patients similar to T1D and others indistinguishable from T2D [8]. There are variations in the phenotype of LADA between populations. For example, populations from Europe, the United Arab Emirates, and Yemen have intermediate features between T1D and T2D [9,10,11]. Nonetheless, in Chinese populations, LADA has similar insulin resistance compared to T2D, and less serum GADA levels [12,13]. The variations in the phenotype of LADA suggest that differences between populations could be attributed to genetic risk factors. In this sense, LADA has genetic traits that interact with the environmental factors of T1D and T2D [6]. The genetic risk factors together with non-genetic factors of LADA could influence the rate of β-cell destruction and thus lead to a heterogenous clinical presentation [14]. Our knowledge is that only one genome-wide association study (GWAS) of LADA has been conducted in a European population; in this study, they found 5 polymorphisms in LADA (genes *HLA-DQB1*, *PTPN22*, *INS*, and *SH2B3*), and these single nucleotide polymorphism (SNP) are related to T1D [15]. In the Chinese Han population, Song et al. (2024) associated LADA and T1D with an *RPS26* variant in a case-control study [16]. On the other hand, Mendelian randomization studies in the European population associated lifestyle risk factors in LADA with risk genotypes related to T2D (*FTO* and *TCF7L2*) [17,18]. In this sense, more studies are necessary to comprehend the genetics of LADA better. However, information about risk genotypes of LADA in non-European populations, including Latin American populations, is scarce. Genetic studies in the Latin American population could reveal LADA risk factors unique to this population. Therefore, this study aims to perform a GWAS with a phenome-wide association study (PheWAS) of LADA of a population of patients with LADA from a hospital in southeastern Mexico and a biobank of the Mexican population.

## 2. Results

A total of 51 phenotypical features were evaluated in our PheWAS analysis, which identified 981 SNPs associated with these phenotypes (*p* < 5 × 10^−5^). The associations are provided in Supplementary Table S1.

In the case-control GWAS analysis for LADA and population-based controls, no genome-wide significant association was found. Still, we observed 8 single-nucleotide polymorphisms associated at the nominal level (*p*-value < 1 × 10^−5^) (Supplementary Figure S1). Five SNPs are intronic in the *LIFR* and *HDAC9* genes, two are intergenic variables, and one is located downstream of the *TMEM20* gene (Table 1).

In LADA cases (*n* = 50), we associated GADA levels and one polymorphism with genome-wide significance (*p* < 5 × 10^−8^). Table 2 resumes the nominal significant variants located in the genome-wide significant locus in chromosome 12. The other nominally significant variants are shown in Supplementary Table S1. Likewise, Table 3 presents nominal significant polymorphisms associated with other phenotypic features in patients with LADA (diabetes onset, height, and hypertension onset).

**Figure 1:**
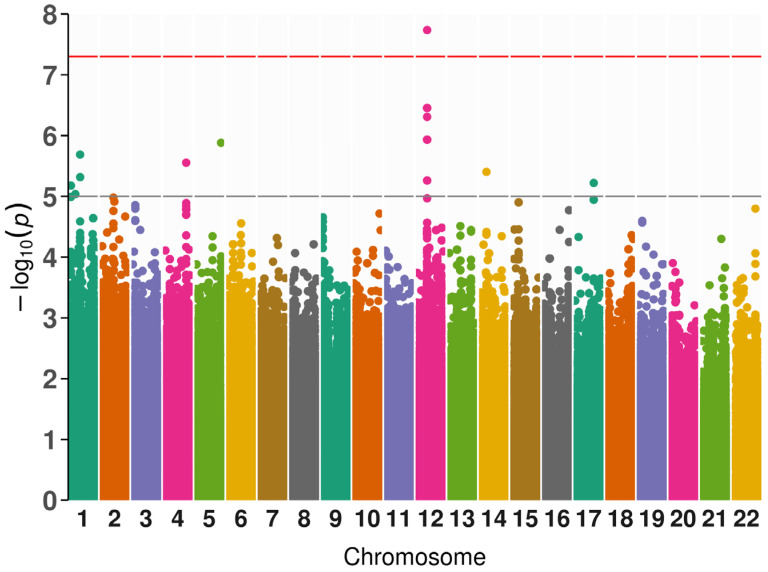
Manhattan plot of the genome-wide association of GADA in patients with LADA. The gray line marks the threshold for nominal significance. Nominal significance was defined as 5 (−log_10_(1 × 10^−5^)). The red line marks the threshold for genome-wide significance and was defined as 7.3 (−log_10_(5 × 10^−8^)).

**Figure 2:**
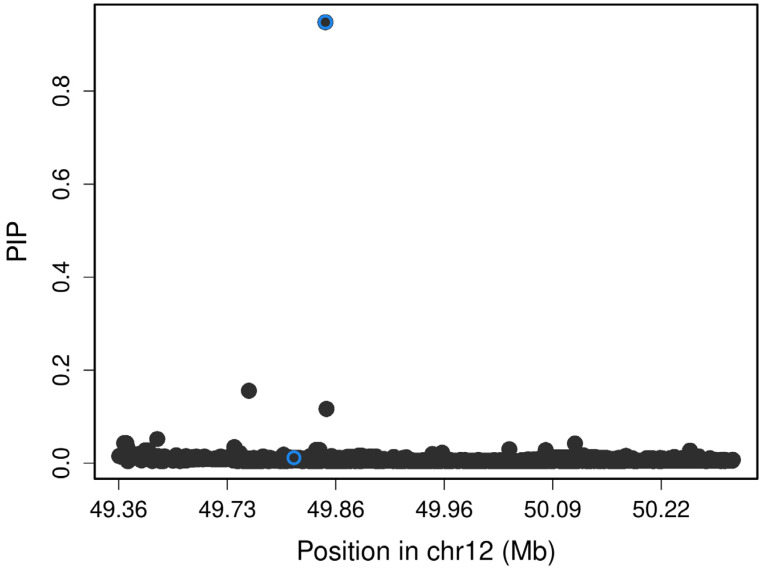
Fine mapping of the locus of rs7305229. The superior blue circle marks rs7305229, which had the highest posterior inclusion probability (PIP) of being the causal variant of the GADA levels phenotype (94.8%).

The SNP is rs7305229, a downstream variant of the *FAIM2* gene (*p* = 1.84 × 10^−8^, β = −1.27, SE = 0.18, and MAF = 0.22). Figure 1 includes the Manhattan plot for the GADA genome-wide associations. After, we performed a fine mapping of the rs7305229 locus, including all SNPs within 1 Mb. Figure 2 shows that the polymorphism rs7305229 had a 94.8% posterior inclusion probability of being causal for the GADA levels phenotype, the greatest posterior probability.

We realized a PheWAS with the GWAS Atlas database, which found 41 traits of the metabolic domain associated with rs7305229 (*p* < 5 × 10^−8^). Bioelectrical impedance-related measures and body mass index were frequently associated with rs7305229. Other traits related to this SNP were salt consumption, age at menarche, and bone mineral density. Notably, we did not find an association between rs7305229 and the immunological domain (*p* > 1 × 10^−5^) (Supplementary Table S2).

The rs7305229 was associated with GADA levels in our population and with body mass index (BMI) in the GWAS Atlas database. Thus, we performed a conditional analysis to identify possible dependent associations. The linear model between the normalized values of both traits showed no association (R^2^ = 0.026, *p* = 0.262). Additionally, the second genome-wide analysis displayed changes in comparison to the first analysis (*p* = 6.97 × 10^−8^, β = −1.26, SE = 0.19).

## 3. Discussion

We explored GWAS associations in multiple phenotypes in individuals diagnosed with LADA from Southern Mexico, and we showed that rs7305229 is associated with GADA antibody levels.

In the comparison between LADA cases and population-based controls, we identified associations with the *LIFR* (leukemia inhibitory factor receptor) gene. These associated SNPs of *LIFR* are intronic variants, and they probably change expression levels rather than alter their protein structure. In addition, LIFR is a transmembrane receptor that binds to IL-6 family members, including the leukemia inhibitory factor and oncostatin-M [19]. LIFR is involved in lipolysis, adipose inflammation, hepatic triacylglycerol accumulation, and insulin resistance in mouse models [20,21]. The existing literature remarks that 56% of patients with T2D have non-alcoholic fatty liver disease (NAFLD) [22]. In this context, the changes in LIFR expression could be related to susceptibility to NAFLD and insulin resistance in patients with LADA, and the LIFR/JAK/STAT3 inflammatory pathway enhances adipocyte lipolysis [21]. Although, the evidence on subjects with LADA is scarce; a study of hospitalized patients in China reported that 24.6% of individuals with LADA had NAFLD [23]. In our population, we found fewer patients with LADA and NAFLD than with T2D and NAFLD; nonetheless, the risk for NAFLD in patients with LADA could be greater than in healthy subjects [24].

We associated a total of 981 SNP with nominal associations in different phenotypes. The phenotypes with more associations were the age of hypertension onset (n = 244), BMI (n = 88), insulin doses (n = 74), and cholesterol levels (n = 67). Notably, these phenotypes resemble the metabolic syndrome criteria [25]. In addition, the LADA genetic constitution has polymorphisms associated with metabolic syndrome [14,15].

Our PheWAS identified a GWAS significant association with a 3′UTR genetic variant (rs7305229) located downstream of the *FAIM2* gene (Fas apoptotic inhibitory molecule 2) with plasmatic GADA antibody levels in patients diagnosed with LADA. Polymorphisms of *FAIM2* are associated with childhood obesity, autoimmune thyroid disease, and obsessive-compulsive disorder [26,27,28]. Additionally, *FAIM2* expression is associated with lymphocyte CD8+ antitumor activity. The FAIM2 protein protects from cell death induced by Fas and plays an important role in calcium balance in the endoplasmic reticulum [29]. Concerning that, FAIM2 KO mouse models showed increased apoptosis of newly activated T lymphocytes. This evidence suggests that FAIM2 protects the T cells from apoptosis, as the first days of a cytotoxic response are hostile for memory T cells [30]. There is limited evidence on the effect of genetic variations of *FAIM2* on GADA antibodies. The GADA antibodies are considered markers of autoimmune activity, which also is mediated by T lymphocytes [14]. Additionally, a pathway analysis of *FAIM2* and its co-expressed genes found that this gene is associated with T cell activation, lymphocyte mediated immunity, and antigen processing and presentation [29]. In addition, CD4 and CD8 T cells have the highest levels of expression of *FAIM2* compared to other cells of the immune system [30]. In this sense, GADA is present in other autoimmune disorders. Furthermore, these subjects with GADA can develop T1D [31,32]. Notably, a study associated a SNP of *FAIM2* with antibody levels in patients with Graves’ disease and Hashimoto’s thyroiditis [28].

We employed the GWAS Atlas data to perform a second PheWAS to compare our observations with previous results from other populations. Notably, this second PheWAS showed that SNP rs7305229 has not been associated with immunologic traits. Instead, this SNP is mainly associated with metabolic traits. In this sense, other variants of *FAIM2* in the 3′UTR region are associated with obesity and body adiposity in other GWAS [33,34]. Nevertheless, we did not find associations between BMI with GADA and rs7305229. Littleton et al. (2024) propose that subjects with fewer anorexigenic POMC neurons would have reduced expression of *FAIM2*, inducing increased appetite, and increasing the risk of becoming overweight in childhood [27]. Interestingly, evidence shows that childhood obesity genetics are a risk factor for diabetes regardless of autoimmunity or insulin resistance [35]. In addition, high BMI is a risk factor for childhood type 2 diabetes and latent autoimmune diabetes in youth [36]. Furthermore, obesity in childhood is also a risk factor in adulthood for T2D and LADA [37].

We hypothesize that our subjects with LADA and rs7305229 would have lower GADA antibody levels. Likewise, other SNPs of the *FAIM2* gene could produce increased BMI and thus, a high risk of diabetes. BMI and GADA antibody levels are heterogeneous features among patients with LADA; some individuals have high antibody levels and a low BMI, while others have low antibody levels and a high BMI [14]. In this regard, Caucasian patients with LADA have a greater proportion of high antibody levels than Chinese subjects with LADA. Conversely, metabolic syndrome is more frequent in the Caucasian population than in Chinese individuals. Another difference is insulin resistance; LADA has similar resistance to T2D in the Chinese population, but in Caucasian patients, it is less pronounced than in T2D [12]. Our population showed low antibody levels, a high frequency of metabolic syndrome, and insulin resistance like T2D. The variability of clinical features of LADA would reflect the differences in genetic and environmental risk factors among individuals and populations [14].

We know this is the first GWAS of LADA performed within the Mexican population; nevertheless, it has some limitations. First, all subjects with LADA were from a regional referral hospital in southeastern Mexico. In addition, the healthy subjects’ genotypes were from a nationwide database, which limited the comparison between phenotypical features. The sample size of our study is small, and we do not have a replication cohort, reducing our statistical power. Therefore, the low statistical power limited the PheWAS analysis to continuous variables only. Although using the subpopulation from southeast Mexico limited the sample size, our GWAS was well controlled, as reflected in our lambda values. In addition, our results could not apply to other populations of Latin America or the world. Allelic frequencies of rs7305229 show variations between local ancestries of Admixture American populations, with Amerindigenous ancestry showing lower allelic frequency in comparison with European and African ancestries (Supplementary Table S3). This evidence suggests that more studies with larger sample sizes about the genetic constitution of LADA are necessary, including studies of other Latin American populations.

## 4. Materials and Methods

### 4.1. Sample Population

We included a subsample of 59 deeply phenotyped individuals diagnosed with LADA from a previous study [24]. These subjects attended the Diabetes Clinic of the Hospital Regional de Alta Especialidad “Dr. Gustavo A. Rovirosa Pérez”, located in Villahermosa, Tabasco. This Diabetes Clinic cares for 505 patients, as the hospital is a referral center for the southeastern states of Mexico, which include Chiapas, Veracruz, and Tabasco. A diabetologist evaluated all recruited subjects from January 2020 to May 2021. We included individuals who met the following criteria: Mexican descent, older than 30 years at the time of diabetes diagnosis, more than six months without insulin treatment after diagnosis, and positivity to GADA determined by ELISA (≥5 UI/mL). Exclusion criteria were patients with a diagnosis of T1D and those less than one year with a diabetes diagnosis; patients without a blood sample were removed. We followed the ethical principles from the Declaration of Helsinki. This study was approved by the Ethics Committee of the High Specialization Regional Hospital “Dr. Gustavo A. Rovirosa Pérez” (HR/ENS/ARM/8073/2023; 5 July 2023). They received verbal and written information about this study. Every patient participated voluntarily without receiving remuneration and signed an informed consent.

We also included a population-based control sample from the Mexican Genomic Database for Addiction Research (MxGDAR/Encodat) [38]. Creation of the MxGDAR/ENCODAT database was approved by the Research Ethics Committees of the Instituto Nacional de Psiquiatría Ramón de la Fuente Muñiz (CEI/C/083/2015) and the Instituto Nacional de Medicina Genómica (01/2017/I). This study was derived from the Mexican National Survey of Tobacco, Alcohol, and Drug Use. Inclusion criteria were Mexican subjects aged 18 to 65 years from the 32 states of Mexico, and with signed informed consent. Individuals from regions where more than 50% of the population speaks a Native American language were excluded. We removed individuals with diabetes diagnoses for our analysis. We included 3121 individuals from this database.

### 4.2. Phenotypic Characterization

A structured questionnaire was applied to recruited patients with LADA. We collected sociodemographic data (age, gender, education, marital status, and socioeconomic status). Family history, pharmacotherapy, and age at diabetes and hypertension diagnosis were retrieved from clinical records. Diabetes complications were registered, including non-alcoholic fatty liver disease, diabetic kidney disease, retinopathy, neuropathy, and cardiovascular complications. Likewise, comorbidities like hypertension, dyslipidemia, and alcohol and tobacco use were evaluated. We measured blood pressure, weight, height, BMI, waist circumference, and hip circumference. Fasting glucose, total cholesterol, HDL cholesterol, LDL cholesterol, and triacylglycerols were determined from the blood serum. GADA antibody levels were detected with an ELISA kit (MyBioSource, San Diego, CA, USA). The total of phenotypical features evaluated was 51.

### 4.3. Microarrays Analysis

We extracted DNA from whole blood samples using a salting-out procedure with the Puregene Blood kit (Qiagen, Germantown, MD, USA) following the manufacturer’s instructions. DNA quality and integrity were evaluated with a Nanodrop spectrophotometer (Thermofisher, Waltham, MA, USA). Samples with 260/280 and 260/230 ratios > 1.8, concentration > 50 ng/µL, and no signs of degradation were used. The DNA was hybridized with the Infinium Psycharray Beadchip (Illumina, San Diego, CA, USA) following the automated protocol. All genotyping procedures were performed in the Unidad de Alta Tecnología para Expresión y Microarreglos of the Instituto Nacional de Medicina Genómica. The microarray analysis was similar to the population-based controls from the MxGDAR database, as previously described [38].

### 4.4. Genotype Quality Control

Quality control of genotypes was performed with PLINK 1.9 version b7.1 [39]. We excluded genetic variants with a call rate < 95%, minor allele frequency (MAF) < 05, *p*-value < 1 × 10^−10^ for a chi-squared test for Hardy–Weinberg equilibrium for cases and *p* < 1 × 10^−6^ for controls, and chimeric alleles (AT and CG). Individuals were excluded if they had a call rate < 95% for all the genetic variants and ±3 standard deviations from the mean heterozygosity rate. We calculated the identity-by-state of all sample pairs to identify genetic relatedness. Pairs of individuals with pi-hat > 0.2 were selected, and related individuals with lower genotyping rates were excluded. Using this threshold, we kept 59 individuals diagnosed with LADA and 3086 population-based controls for further analysis.

### 4.5. Population Stratification

We used the pcair function of the GENESIS package version 2.30 for R version 4.2 to estimate principal components analysis (PCA) to evaluate population stratification. We included independent SNPs filtered by linkage disequilibrium pruning (threshold: 0.1, slide size: 106). Nonetheless, the population analysis showed the previously reported differences between the Southeast and other regions of Mexico [40]. As previously described, the population from the south of Mexico could have a genetic structure different from that of the country’s center [38]. To avoid effects in our statistical tests for population stratification, we clustered cases and controls. We visualized the potential clustering of subpopulations inside our sample with Uniform Manifold Approximation and Projection (UMAP) using the first 5 principal components (PCs). Two clusters were observed: one group with individuals from various regions of Mexico (cases n = 12, controls n = 2592), and the other with subjects mainly from southeastern Mexico (cases n = 50, controls n = 491). The subpopulation that had the most cases from our LADA sample was selected (Supplementary Figure S2).

### 4.6. Genotype Imputation

We performed the imputation of autosomal genetic variants using the TOPMed Imputation Server with the TOPMed-r2 reference panel [41]. We used the following parameters as post-imputation quality control, keeping those genetic variants with MAF > 0.05, quality score (R^2^) > 0.7, and chi-squared *p* > 1 × 10^−6^ for the Hardy–Weinberg equilibrium. After quality control, we kept 5,797,363 genetic variants for the following analysis.

### 4.7. Phomene-Wide Association Study (PheWAS)

The GENESIS package for R was employed to perform a case-control GWAS of LADA (n = 50) versus controls (n = 491), including the first 5 PCs, sex, and age as covariates. The statistic inflation factor (λ) was 1.082. Additionally, we performed genome-wide linear regressions between SNPs and phenotypic features of LADA cases with PLINK 1.9. Covariates for this analysis were the first 2 PCs, sex, and age. Included phenotypes were continuous variables (anthropometric measures, biochemical measures, antibody levels, and years of disease evolution). Categorical variables were excluded from PheWAS as they had poor quality control results. A *p*-value < 5 × 10^−8^ was considered genome-wide significant and a *p*-value < 1 × 10^−5^ was nominally significant for both analyses. QQ plots and Manhattan plots were realized with ggplot2 and fastman libraries for R.

### 4.8. Fine Mapping

We used the susieR package version 0.12.35 for R version 4.2 to perform a fine mapping for the associated loci using the summary statistics that showed GWAS significance. A 1000 kb window around the top genetic variant was selected as a significant locus. The LDlinkR package for R was employed to acquire the LD correlation matrix from the LDlink web application, and we used the Mexican Ancestry from Los Angeles (MXL) population as a reference [42]. Genetic variants with 95% posterior inclusion probability (PIP) were considered part of the credible set.

### 4.9. PheWAS Analysis

To find associations of SNPs with phenotypes in previous GWAS, the GWAS Atlas website was employed for PheWAS analysis of the nominally significant SNP. Associations with *p*-value < 5 × 10^−8^ were considered significant.

### 4.10. Conditional Analysis

We realized a conditional analysis to identify phenotypes dependently associated with significant GWAS loci. For patients with LADA, a second genome-wide analysis was performed with PLINK 1.9 [43]. This analysis included body mass index as a covariant for the significant GWAS loci. Also, a linear model for body mass index and GADA levels was calculated with R.

## 5. Conclusions

We performed a phenome-wide association study of LADA in subjects from southeastern Mexico, and the results indicated that rs7305229 was associated with GADA antibody levels. These findings contribute to understanding the genetic mechanisms underlying LADA, as its heterogeneity among individuals may be reflected in their genetic constitutions. Because the genetic risk factors for LADA may vary among populations, it is necessary to expand research in this area.

## Figures and Tables

**Table 1:** Significant variants associated with LADA.

Gene	Effect	rsID	Chr	Position	Alleles	MAF Cases	MAF Controls	R^2^	β	SE	*p*
*LIFR*	Intron	rs1012760	5	38579223	T/C	0.42	0.18	0.96	1.06	0.23	8.12 × 10^−6^
*LIFR*	Intron	rs10040852	5	38580639	A/G	0.42	0.18	-	1.06	0.23	8.12 × 10^−6^
*LIFR*	Intron	rs6883795	5	38582906	A/C	0.42	0.18	0.96	1.06	0.23	8.74 × 10^−6^
*LIFR*	Intron	rs6862038	5	38596366	A/G	0.41	0.17	0.95	1.05	0.23	8.35 × 10^−6^
*HDAC9*	Intron	rs17419156	7	18363323	T/G	0.45	0.33	0.79	1.15	0.24	1.11 × 10^−6^
*-*	Intergenic	rs11770918	7	68043584	A/G	0.25	0.07	0.9	1.44	0.30	2.62 × 10^−6^
*-*	Intergenic	rs73144105	7	68047584	C/T	0.25	0.07	0.9	1.44	0.30	2.62 × 10^−6^
*TMEM260*	Downstream	rs17091924	14	56655475	G/T	0.45	0.27	0.87	1.02	0.22	5.65 × 10^−6^

Alleles are reference/other. All variants were imputated (except rs10040852), R^2^ value are from imputation. β, SE, and *p* values are from the genome-wide association.

**Table 2:** Significant variants associated with GADA levels in patients with LADA (n = 50).

rsID	Chr	Position	Alleles	MAF Cases	MAF GnomAD	R^2^	β	SE	*p*
rs7316671	12	49846521	G/A	0.21	0.44	0.83	−1.26	0.21	3.52 × 10^−7^
rs7316688	12	49846558	G/C	0.21	0.39	0.84	−1.26	0.21	3.52 × 10^−7^
rs11169176	12	49847230	G/A	0.21	0.35	0.84	−1.26	0.21	3.52 × 10^−7^
rs4898538	12	49849588	G/A	0.34	0.4	0.81	−1.09	0.19	1.17 × 10^−6^
rs7299134	12	49849889	A/G	0.21	0.35	0.84	−1.26	0.21	3.52 × 10^−7^
rs11169178	12	49850240	C/T	0.34	0.41	0.81	−1.09	0.19	1.17 × 10^−6^
rs7303074	12	49850241	G/A	0.21	0.36	0.83	−1.26	0.21	3.52 × 10^−7^
rs7302855	12	49850260	C/T	0.21	0.35	0.84	−1.26	0.21	3.52 × 10^−7^
rs10875980	12	49850984	A/T	0.19	0.35	0.84	−1.22	0.24	5.48 × 10^−6^
rs10875981	12	49851185	G/C	0.34	0.4	0.83	−1.09	0.19	1.17 × 10^−6^
rs10875982	12	49851923	A/G	0.19	0.35	0.85	−1.22	0.24	5.48 × 10^−6^
rs11169182	12	49854269	C/T	0.19	0.36	0.85	−1.22	0.24	5.48 × 10^−6^
rs11169183	12	49854299	T/C	0.34	0.41	0.82	−1.09	0.19	1.17 × 10^−6^
rs11169184	12	49854562	T/A	0.34	0.41	0.82	−1.09	0.19	1.17 × 10^−6^
rs7953118	12	49854941	T/C	0.34	0.41	0.82	−1.09	0.19	1.17 × 10^−6^
rs67906820	12	49855087	TC/T	0.2	0.36	0.84	−1.22	0.21	4.93 × 10^−7^
rs7306760	12	49856886	C/T	0.34	0.43	0.82	−1.09	0.19	1.17 × 10^−6^
rs7305229	12	49866481	C/T	0.22	0.36	0.83	−1.27	0.18	1.84 × 10^−8^*

Alleles are reference/other. All variants were imputated, R^2^ value are from imputation. β, SE, and *p* values are from the genome-wide association. * = genome-wide significance.

**Table 3:** Significant variants associated with different phenotypes in patients with LADA (n = 50).

Phenotype	rsID	Chr	Position	Alleles	MAF Cases	MAF GnomAD	R^2^	β	SE	*p*
Hypertension onset	rs62060292	16	59074272	G/A	0.14	0.15	0.96	−1.75	0.18	6.18 × 10^−8^
Diabetes onset	rs146135680	3	118172739	C/G	0.10	0.11	0.75	−1.41	0.22	6.48 × 10^−8^
Diabetes onset	rs79720909	3	118168643	C/T	0.11	0.14	0.76	−1.37	0.21	7.82 × 10^−8^
Height	rs12415892	10	104517955	T/C	0.26	0.35	0.96	0.99	0.15	9.30 × 10^−8^
Height	rs10709652	10	104518782	GT/G	0.26	0.41	0.95	0.99	0.15	9.30 × 10^−8^
Height	rs11192097	10	104519244	C/G	0.26	0.35	0.95	0.99	0.15	9.30 × 10^−8^
Height	rs10884013	10	104519545	G/A	0.26	0.35	0.95	0.99	0.15	9.30 × 10^−8^
Height	rs4918104	10	104519664	G/A	0.26	0.34	0.95	0.99	0.15	9.30 × 10^−8^

Alleles are reference/other. All variants were imputated, R^2^ value are from imputation. β, SE, and *p* values are from the genome-wide association.

## Data Availability

The datasets of the MxGDAR/ENCODAT database can be found in the European Variation Archive (EVA) with the ID PRJEB37766. The original contributions presented in the study are included in the article/Supplementary Material, further inquiries can be directed to the corresponding author/s.

## Supplementary Materials

The following supporting information can be downloaded at: https://www.mdpi.com/article/10.3390/ijms251810154/s1.
