# Supplementary material for: Phenome-Wide Association Study of Latent Autoimmune Diabetes from a Southern Mexican Population Implicates rs7305229 with Plasmatic Anti-Glutamic Acid Decarboxylase Autoantibody (GADA) Levels
Source: Int J Mol Sci. 2024 Sep 21;25(18):10154. doi: 10.3390/ijms251810154 (PMC11432505; doi:10.3390/ijms251810154)
Supplement: Supplementary file 1 [file ijms-25-10154-s001.zip › Supplementary Figure S1.pdf]

Supplementary Figure 1. Manhattan plot of the GWAS of LADA.

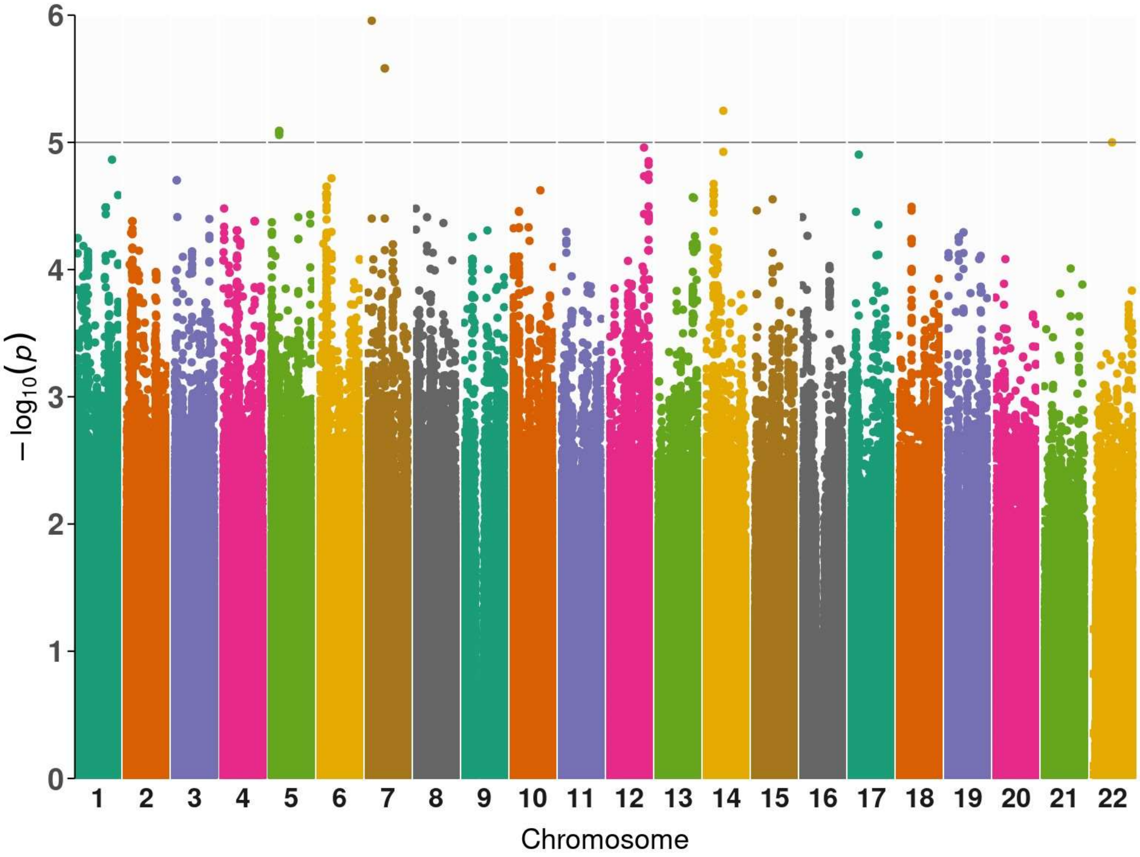

The gray line marks the threshold for nominal significance. Nominal significance was defined as 5 ($-\log_{10}(1x10^{-5})$).
